# Multi-criteria decision analysis: technique for order of preference by similarity to ideal solution for selecting greener analytical method in the determination of mifepristone in environmental water samples

**DOI:** 10.1007/s11356-024-32961-3

**Published:** 2024-04-05

**Authors:** Tlou A. Makwakwa, Dineo E. Moema, Titus A. M. Msagati

**Affiliations:** 1 Department of Chemistry, College of Science, Engineering and Technology, University of South Africa, Johannesburg, 1709 Florida South Africa; 2 Institute for Nanotechnology and Water Sustainability, College of Science, Engineering and Technology, University of South Africa, Johannesburg, 1709 Florida South Africa

**Keywords:** Mifepristone, MCDA–TOPSIS, Analytical methods, Green analytical chemistry, Metrics tools, Correlation matrix

## Abstract

This work proposes the use of multi-criteria decision analysis (MCDA) to select a more environmentally friendly analytical procedure. TOPSIS, which stands for Technique for Order of Preference by Similarity to Ideal Solution, is an example of a MCDA method that may be used to rank or select best alternative based on various criteria. Thirteen analytical procedures were used in this study as TOPSIS input choices for mifepristone determination in water samples. The input data, which consisted of these choices, was described using assessment criteria based on 12 principles of green analytical chemistry (GAC). Based on the objective mean weighting (MW), the weights for each criterion were assigned equally. The most preferred analytical method according to the ranking was solid phase extraction with micellar electrokinetic chromatography (SPE-MEKC), while solid phase extraction combined with ultra-high performance liquid chromatography tandem mass spectrometry (SPE-UHPLC-MS/MS) was ranked last. TOPSIS ranking results were also compared to the green metrics NEMI, Eco-Scale, GAPI, AGREE, and AGREEprep that were used to assess the greenness of thirteen analytical methods for mifepristone determination. The results demonstrated that only the AGREE metric tool correlated with TOPSIS; however, there was no correlation with other metric tools. The analysis results suggest that TOPSIS is a very useful tool for ranking or selecting the analytical procedure in terms of its greenness and that it can be easily integrated with other green metrics tools for method greenness assessment.

**Supplementary Information:**

The online version contains supplementary material available at 10.1007/s11356-024-32961-3.

## Introduction

**Fig. 1:**
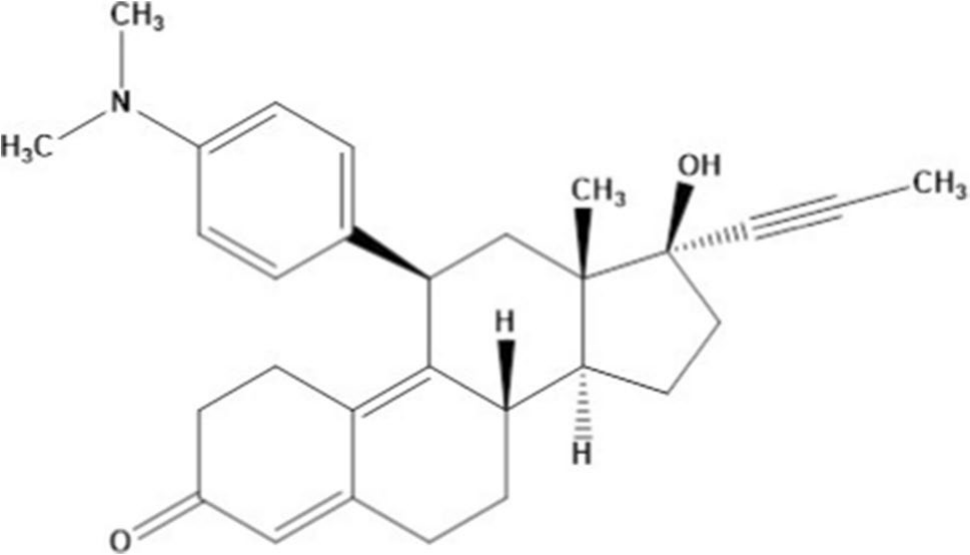
Molecular structure of mifepristone

Mifepristone (Fig. 1) is an antiprogestational steroid that also functions as an oral contraceptive, an abortifacient, and a hormone antagonist (Ault et al. 2023). Mifepristone is extensively metabolized after oral administration by demethylation and hydroxylation, with the early metabolic stages catalysed by the cytochrome P450 (CYP) enzyme CYP3A4 (Heikinheimo et al. 2003). The three most proximal metabolites of mifepristone are monodemethylated, didemethylated, and hydroxylated metabolites. They all retain significant affinity toward the human progesterone and glucocorticoid receptors; additionally, serum concentrations of these three metabolites are in the same range as those of the parent drug (Heikinheimo 1997). The plasma half-life of mifepristone has been found to range between 24 and 48 h when measured using high performance liquid chromatography and between 55 and 90 h when measured using RIA or radioreceptor assays (Johanssen and Allolio 2007). When compared to other steroids, which have plasma half-lives that range from minutes (progesterone) to 3–5 h, RU 486 exhibits an unusual lengthy plasma half-life; however, these values were most likely affected by the presence of cross-reacting metabolites such as dexamethasone (Johanssen and Allolio 2007). This property can also be accounted for by the drug’s strong binding (Johanssen and Allolio 2007). Mifepristone residues in the aquatic environment have recently grown to be one of the most alarming public health concerns. Mifepristone has the potential to be hazardous to aquatic’s life and humans, and it has been linked to the fast growth of endocrine disruptors in the environment (Fabbrocini et al. 2019).

Mifepristone is widely distributed in the environment, and several studies that have been published using various analytical techniques demonstrate the ongoing interest in and intense level of research effort on this compound’s presence in the environment. Among these approaches, chromatographic techniques and other extraction techniques are well known for using hazardous solvents and having a negative impact on the environment (Chanduluru and Sugumaran 2022). Significant efforts have recently been made to determine the quantities of organic pollutants in environmental matrices, with a focus on environmentally friendly processes and the development of the so-called green analytical chemistry (GAC) methods (Farré et al. 2010). GAC is one of the most active fields of green chemistry research and development, and it presents a significant challenge for environmental analytical chemists (Korany et al. 2017). GAC’s goal is to introduce innovative techniques and methodologies that reduce environmental and occupational dangers at all stages of chemical analytical procedures, allowing for faster and more energy-efficient processes without affecting method performance (Korany et al. 2017). The procedures used to establish if an analytical procedure can be classified as green should be standard and reliable. Such processes should be compared, validated, and used as the primary parameter in the development of green analytical methods. A systematic evaluation of green metrics tools in a real-world analytical chemistry application is critical for selecting the most environmentally friendly procedure. Although suitable analytical methods for quantifying mifepristone in various environmental matrices have been established, none of these approaches have been evaluated for their greenness. Even though the greenness of these methods should be evaluated, there is currently a scarcity of well-defined and validated green analytical procedures that are distinguished by both good analytical and environmental performance. Given these circumstances, evaluation of the greenness of the analytical techniques reported for mifepristone determination is critical for selecting the most environmentally friendly one to conserve the environment. Although there are several analytical methods available, selecting one that is adequate, sensitive, and environmentally friendly can be a challenging undertaking because many different factors need to be considered (Płotka-Wasylka and Wojnowski 2021). The quantity of waste produced, the toxicity and environmental impact of all chemicals used, the waste generated, the energy consumption used in the analytical process, and the safety of the analytical method are a few examples of such factors (Turner 2013). The selection can therefore be backed up by multicriteria decision analysis.

An example of multicriteria decision analysis (MCDA) tool, Technique for Order of Preference by Similarity to Ideal Solution (TOPSIS), has emerged as a green alternative tool that can incorporate greenness assessment into analytical procedures and has been used to identify the best alternative from a variety of potential options (Bigus et al. 2016). The TOPSIS approach is straightforward and relatively simple, because it uses an algorithm whose solution process does not change irrespective of the number of decision criteria and alternatives (Emovon and Aibuedefe 2020). In this context, TOPSIS becomes a critical component tool that enables ranking of the procedures. The most prevalent metrics tools used to analyse the greenness of analytical procedures are National Environmental Methods Index (NEMI), Eco-Scale Assessment (ESA), Green Analytical Procedure Index (GAPI), and Analytical GREEnness metric (AGREE) (Gamal et al. 2021). These tools are critical because they evaluate risks, ensure that environmental and human factors are protected from chemical harm, and provide guidelines for developing new or improving existing methods (Sajid and Płotka-Wasylka 2022). Considering the foregoing observations, this paper reports systematically on the use of multicriteria decision analysis and green metrics tools to select and evaluate the greenness of analytical procedures for mifepristone determination in water samples. The study’s objectives were to (a) select the greenest analytical procedure using TOPSIS approach; (b) to evaluate and compare the greenness of various analytical methods developed for determination of mifepristone using the Green Analytical Procedure Index (GAPI), Eco-Scale, GAPI, AGREE, and AGREEprep tools; and (c) to establish correlations between TOPSIS and metrics tools.

## Methodology

In this study, the TOPSIS strategy for selecting the greener analytical method for mifepristone determination in water is provided. The underlying TOPSIS algorithm has been well discussed elsewhere in the literature (Abdal 2020). As a result, the primary focus in this section will be on the main concepts and steps, with mathematical detail provided in the supplementary section (Eqs. 1–8). The general scheme of the TOPSIS approach was carried out in the following steps, as indicated in Table 1. Briefly, the set of alternatives is identified after defining the decision problem. The important characteristics, i.e., criteria, must then be defined, along with their relative relevance by assigning weights to the criteria. The key decision criteria were selected in accordance with the 12 principles of green analytical chemistry. These criteria include the use of direct analytical techniques, the use of small sample size, in situ measurements, step numbers, automation and miniaturization, derivatization, waste generation, multianalyte, energy minimization, renewable reagents, toxic reagents, and safety for operator (Mazzaracchio et al. 2022). GAC principles were translated into 0–1 AGREE calculator scores (default weighting) using the input data for the 12 decision criteria. These scores were then used as inputs in the TOPSIS decision matrix (Supplementary Table S1). In the real world, for example, because of limited or unavailable information, human judgements, including preferences, are often ambiguous and cannot be estimated using exact numerical data, which is often not so deterministic (Jahanshahloo, Lotfi, and Izadikhah 2006). As an advantage of this approach, the use of AGREE scores becomes crucial to attaining a fair evaluation process of the reported methods. The concept of “the higher the better’’ was applied to all preference functions for all criteria. A summary of transformations applied to every GAC principle is presented in graphical form elsewhere in the literature (Pena-Pereira et al. 2020). The decision maker’s preferences, which are represented by the weights assigned to the criteria, substantially influence how alternatives are ranked (Tobiszewski and Orłowski 2015a). In most cases, it is preferable to use the weights of specific components to distinguish between the relative relevance of criteria. Effectively, changing weights values can also change the ranking result. When there are no dominant criteria or sufficient information to decide, the weights for all criteria are set to equal (Odu 2019). In Analytical GREEnness Metric approach, equal weights are by default set for all 12 GAC principles (Pena-Pereira et al. 2020). Therefore, a weighting assignment involved equal treatment of all criteria, thereby resulting in a weight of 0.0833 for each criterion. Table 2 summarizes the analytical methodologies considered for TOPSIS analysis, highlighting the importance of mifepristone analyses.

**Table 1:** TOPSIS implementation process

Steps in MCDA-TOPSIS analysis	Description
1. Stating the problem	Selecting the analytical method for mifepristone determination in water
2. Selection of alternatives	Thirteen analytical methods were identified
3. Selection of criteria	Twelve green analytical chemistry (GAC) principles
4. Weighting the criteria	Setting the relative importance of each criterion
5. TOPSIS analysis	Ranking of analytical methods as the result
6. Selecting the best alternative	The first from the ranking

**Table 2:** Analytical methodologies as alternatives in TOPSIS analysis for mifepristone determination

Alternative	Analytical methodology	Abbreviation	Reference
Method 1	Solid Phase Extraction–High Performance Liquid Chromatography Tandem Mass Spectrometry	SPE-HPLC–MS/MS	(K. Zhang and Fent 2018)
Method 2	Solid Phase Extraction–Liquid Chromatography–Atmospheric Pressure Chemical Ionization/ Atmospheric Pressure Photoionization–High Resolution Product Scan Mode	SPE-LC-APCI/APPI-HRPS	(Šauer et al. 2018)
Method 3	Solid Phase Extraction–Liquid Chromatography– Atmospheric Pressure Chemical Ionization/ Atmospheric Pressure Photoionization–High Resolution Product Scan Mode	SPE-LC-APCI/APPI-HRPS	(Golovko et al. 2018)
Method 4	Solid Phase Extraction–Ultra Performance Liquid Chromatography Tandem Mass Spectrometry	SPE-UPLC-MS/MS	(Shen et al. 2018)
Method 5	Square Wave Voltammetry	SWV	
Method 6	Solid Phase Extraction–Liquid Chromatography Tandem Mass Spectrometry	SPE-LC–MS/MS	(X. Liu et al. 2010)
Method 7	Solid Phase Extraction–Micellar Electrokinetic Chromatography	SPE-MEKC	(Rucins et al. 2018)
Method 8	Solid Phase Extraction–Ultra High Performance Liquid Chromatography Tandem Mass Spectrometry	SPE-UHPLC-MS/MS	(S. S. Liu et al. 2014)
Method 9	Solid Phase Extraction–High Performance Liquid Chromatography Tandem Mass Spectrometry	SPE-HPLC–MS/MS	(K. Zhang et al. 2017)
Method 10	Solid Phase Extraction–Ultra High Performance Liquid Chromatography Tandem Mass Spectrometry	SPE-UHPLC-MS/MS	(J. N. Zhang et al. 2021)
Method 11	Solid Phase Extraction–Ultra High Performance Liquid Chromatography Tandem Mass Spectrometry	SPE-UHPLC-MS/MS	(S. S. Liu et al. 2015)
Method 12	Solid Phase Extraction–Liquid Chromatography Tandem Mass Spectrometry	SPE-LC–MS/MS	(Macikova et al. 2014)
Method 13	Solid Phase Extraction–Liquid Chromatography Tandem Mass Spectrometry	SPE-LC–MS/MS	(Ammann et al. 2014)

## Results and discussion

### The ranking with TOPSIS

The TOPSIS algorithm is based on the principle that the chosen alternative should be the closest to the positive ideal solution and the furthest away from the negative ideal solution (Jahanshahloo, Lotfi, and Izadikhah 2006). Table 3 shows the ranking results of analytical methods for mifepristone determination, with SPE-MEKC (method 7) being the greener of the potential alternatives (ranked first). The SPE-UHPLC-MS/MS (method 11) is the least green analytical method, ranking last among all possible methods. Methods 8 and 11 have the same values of relative closeness, as well as the same number and types of analytes determined in a single run, but they use different samples. The same trend was observed with methods 2 and 3, with the exception that they both use the same types of samples. The ranking results also revealed some additional fascinating information. Most studies used SPE coupled to LC–MS/MS, except for SWV (method 5) and SPE-MEKC (method 7). Among these methods, only three methods (1, 2, and 3) were automated. SPE, on the other hand, is a popular sample preparation technique, but it is known to impact negatively on the environment as it requires considerable volume of solvents that are often hazardous and usually produce solid waste (Tobiszewski and Orłowski 2015b). As a result, it is not regarded as an environmentally benign method of sample preparation.

**Table 3:** The TOPSIS ranking of analytical procedures for determining mifepristone in water

Alternative no	Analytical technique	Relative closeness	Ranking
Method 1	SPE-HPLC–MS/MS	0.3031	2
Method 2	SPE-LC-APCI/APPI-HRPS	0.2962	3
Method 3	SPE-LC-APCI/APPI-HRPS	0.2962	4
Method 4	SPE-UPLC-MS/MS	0.2192	6
Method 5	SWV	0.2725	5
Method 6	SPE-LC–MS-MS	0.1830	10
Method 7	SPE-MEKC	0.5877	1
Method 8	SPE-UHPLC-MS/MS	0.1747	12
Method 9	SPE-HPLC–MS/MS	0.1964	9
Method 10	SPE-UHPLC-MS/MS	0.1996	8
Method 11	SPE-UHPLC-MS/MS	0.1747	13
Method 12	SPE-LC–MS/MS	0.1950	11
Method 13	SPE-LC–MS/MS	0.2149	7

In terms of analytical methodologies, both SWV and MEKC procedures have limitations in terms of sensitivity and selectivity when compared to LC–MS/MS methods. Method 5, ranked in position 5, was directly applied for the determination of mifepristone without prior sample preparation, which is a desirable plus factor in terms of GAC principles. While the sensitivity of method 7 is practically less than LC–MS/MS methods, other limitations can be enumerated. A practical limitation of MEKC methodology is its intrinsic difficulty with transitioning CE detectors to MEKC, causing low detection sensitivity (Lian et al. 2014). In addition, MEKC may suffer from poor reproducibility of electroosmotic flow between samples (Santhi et al. 2021). Despite all these limitations, the ranking of SPE-MEKC at position number one is logical since this method is considered green mainly due to its observed low energy and solvent consumption. This observation is somewhat consistent with the concept of GAC, particularly when comparative green assessment studies are drawn between LC–MS/MS and MEKC methods.

The major goal of GAC principles is to minimize or mitigate the negative environmental effects of chemical analysis, while maintaining the traditional analytical characteristics of accuracy, sensitivity, selectivity, and precision (Pena-Pereira et al. 2020). It has already been highlighted that SPE-MEKC (method 7) with a relative closeness value of 0.5877 is by far the greenest method, although it is significantly less sensitive and selective than LC–MS/MS methods. As Nowak and co-workers point out (Nowak et al. 2021), a major difficulty in the implementation of GAC principles is the necessity to correctly balance and reconcile the method’s greenness with its potential usefulness. None of the assessed methods, including method 7, precisely meet the requirement criteria for this critical feature of green chemistry. This means that the environmental effect features of the described analytical methods in this study were most likely not considered during their development and application. As analytical methods are continuously being developed, it is, therefore, advisable that future research regarding mifepristone determination be aimed toward the application and implementation of green analytical principles to protect the environment. Strategies for greening analytical processes have already been reviewed; readers are referred to the work published by Chanduluru and Sugumaran (Chanduluru and Sugumaran 2022) for more information.

### Greenness assessment of the reported analytical methods

The national environmental method index (NEMI), the analytical eco-scale assessment (ESA) method, green analytical procedure index (GAPI), Analytical GREEnness Metric Approach (AGREE), and AGREEprep tool were used to assess the greenness of the described analytical methods for mifepristone determination. The specifics of the metrics under consideration, including their advantages and disadvantages, have been discussed elsewhere in the literature (Gamal et al. 2021; Pena-Pereira et al. 2022). Briefly stated, the Eco-Scale is a point scale grading system where 100 points is the starting point for the ideal green method. Any deviation from the ideal green analysis in this measurement, as determined by the impact of the chemicals and processes used in the analytical procedure parameters, results in the imposition of penalty points and a decrease in this value (Gałuszka et al. 2012). The National Environmental Methods Index (NEMI) (Tobiszewski 2016) and Green Analytical Procedure Index (GAPI) (Płotka-Wasylka and Wojnowski 2021) each use coloured pictograms to symbolically express how environmentally friendly various aspects of the analytical procedure are. The four key terms that make up the condensed NEMI pictogram are persistent bio-accumulative and toxic (PBT), Hazardous, Corrosive, and Waste. Each pictogram quadrant’s colour coding can be green if the method evaluated met the set selection criteria for that quadrant or less green if the quadrant is empty, depending on the method evaluated (Tobiszewski 2016). A GAPI pictogram, on the other hand, is represented as five pentagrams, with the colours green, yellow, and red, respectively, denoting low, medium, and high environmental impact (Płotka-Wasylka and Wojnowski 2021). The Analytical Greenness Calculator (AGREE) (Nowak et al. 2021) uses a simple algorithm that quantitatively expresses the overall greenness of the method on a 0–1 scale, as well as a pictogram that directly refers to the 12 GAC rules. Lastly, AGREEprep is the metric used to assess the environmental impact of sample preparation. The final assessment score is based on ten impact categories that are recalculated in the range of zero to one sub scores. A colourful pictogram representing the assessment’s findings shows how well the sample preparation process performed overall in terms of sustainability (Pena-Pereira et al. 2022).

The graphic presentation of these tools for the reported analytical methods is shown in Table 4. The NEMI tool displayed nearly identical findings since all the methods’ pictograms had three green parts, except for method 5 and method 7, where the former method had just one part that was shaded green. Out of all the reported methods, only method 7 fulfilled the set conditions of green chemistry as all the quarters of the pictogram are coloured green, making it inherently green. Method 5 is distinguished by the generation of excessively high amount of waste, and along with other methods, did not fulfil the greenness conditions. This is primarily owing to the use of hazardous or non-biodegradable reagents or chemicals such as methanol and acetonitrile. These solvents have a detrimental environmental impact, which is why they are included as non-eco-friendly solvents in the toxic release inventory (TRI). When compared to NEMI, the ESA tool presented more detailed semi-quantitative results. By applying this tool, methods 12 and 13 scored highly in the Eco-Scale, both with 90 ESA scores, followed by methods 2 and 3, both with 89 ESA scores. All the methods have a score of 81 or higher, indicating that they are excellent green methods. Methods 12 and 13 are the greenest of the reported methods, simply due to their highest ESA scores. Regarding the GAPI tool, it was observed that none of the reported methods had a pictogram free of a red colour, clearly indicating the weakest points in analytical procedures. Method 5, followed by methods 6 and 7, can be described as the least polluting ones, with six and four green subcategories, respectively. Method 5 was also found to be the only method having a green subcategory 5 related to direct analysis without sample preparation, a condition that is more favourable in terms of green analytical chemistry (Tobiszewski et al. 2009).

**Table 4:**
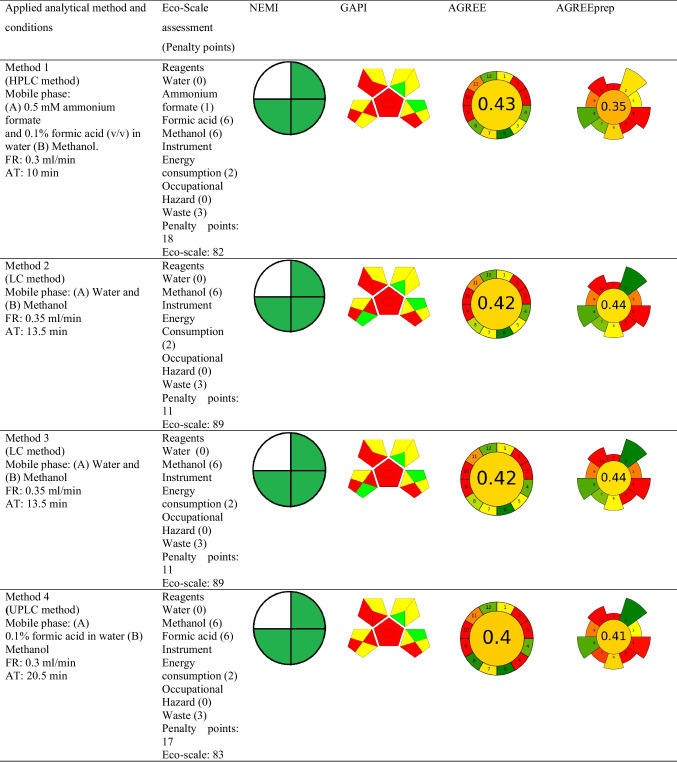

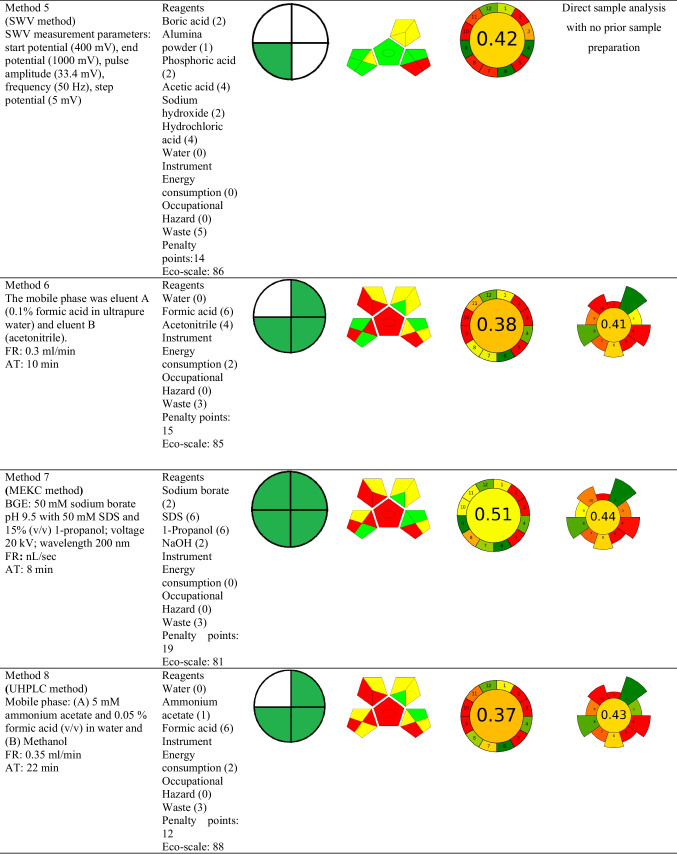

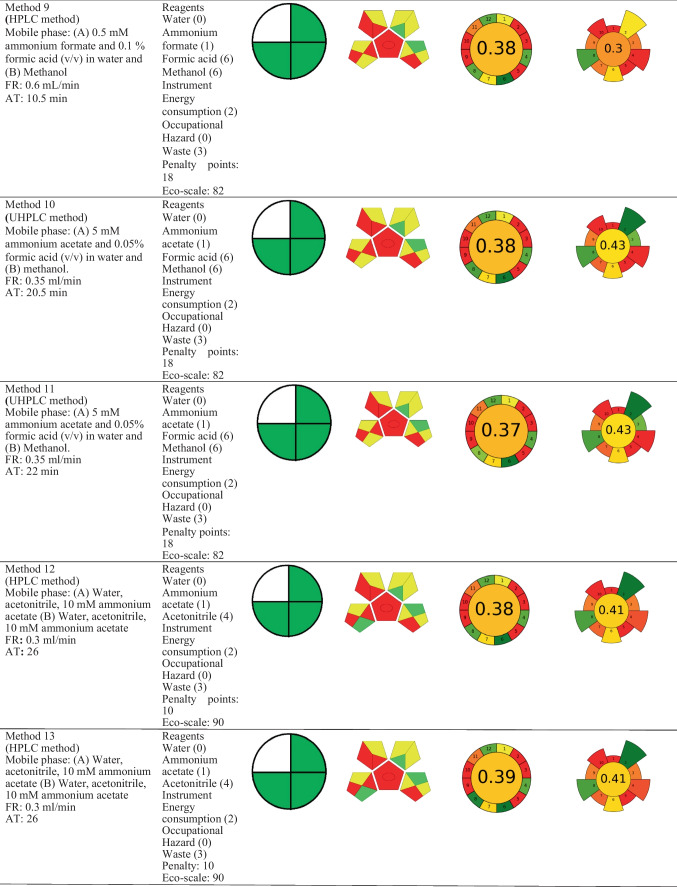
Applied analytical methods and conditions

FR; flow rate, AT; analysis time

The AGREE metric was also explored for a comprehensive green evaluation because it can be used quantitatively to measure all GAC criteria. The AGREE tool scores ranged from 0.37 to 0.5, with SPE-MEKC technique (method 7) scoring higher than the other methods described. However, AGREE metrics indicate that a method must score 0.6 to be considered green (Abdelgawad et al. 2022). Based on this criterion, none of the evaluated methods qualify as green, meaning that they have a negative impact on the environment. Also relevant to the GAC field, sample preparation and extraction procedures are considered the most two polluting parts of analytical method development that can be best adapted to fulfil the principles of green analytical chemistry (Billiard et al. 2020). In this context, AGREEprep becomes one of the most essential tools for evaluating greenness because it provides substantial and in-depth information on sample preparation when comparing different analytical methodologies. It is based on ten categories of green sample preparation, with a scale sub-score ranging from 0 to 1 and is then used to determine the overall assessment score (Wojnowski et al. 2022). The result is represented by a colourful pictogram, with the final evaluation value located inside a coloured circle in the centre of the pictogram, demonstrating the complete sample preparation greenness performance (Martínez et al. 2022). Since SPE was the only applicable extraction technique in the published procedures, AGREEprep was used to evaluate SPE for mifepristone determination in water samples. The pictograms of AGREEprep for evaluated SPE methods, as presented in Table 4, reveal that the methods did not meet most of the criteria and confirm that the reported methods are not environmentally friendly. However, some general observations can be enumerated. According to the parameters for evaluating AGREEprep greenness, methods 2, 3, and 7 had the highest score of 0.44, while the other methods under consideration had scores that were somewhat similar and lower, ranging from 0.30 to 0.43. Additionally, when criteria covering different aspects contributing to the entire sample preparation are considered, certain similarities could be observed. All the reported methodologies have disadvantages, which are mostly related to the use of ex situ sample preparations and the highest number of identified hazards associated with the chemicals used, which could have potentially posed risks to operator’s safety. The reported methods could be upgraded to greener alternatives by choosing processes that minimize extensive sample transportation, using sample preparations that are in integrated in analytical procedures, and minimizing hazards associated with the procedures, including physical ones.

In summary, assessment tools applied in this study can aid in finding weaknesses of the entire analytical procedure and suggest more environmentally friendly options. When the five tools are compared, only the ESA tool classified the reported methods as green ones, though it just offered a quantitative assessment without in-depth explanation of the relevant ecological unfriendly operations of the assessed analytical method. The NEMI tool has shown to be quite ineffective when used alone to assess greenness; other techniques must be added to achieve a precise and accurate assessment. Both GAPI and AGREE tools gave thorough information on the entire analytical procedures by assessing each step of the evaluated methods. AGREEprep, on the other hand, promoted sustainability by evaluating method’s level of sample preparation greenness performance. It is evident that developing new or appropriately adjusting existing methods, as well as minimizing the use harmful organic solvents, will provide safe and environmentally friendly analytical procedures. This is attainable if researchers and analysts avoid using solvents and materials haphazardly in the laboratories. In addition, prior to conducting research, there is a need to thoroughly consider how to develop green methods. It is now possible to model analytical methodologies both theoretically and experimentally, resulting in robust and reliable results. In this regard, chemometrics approach becomes a valuable tool in areas of GAC and may be integrated in the planning of experiments to effectively balance and reconcile the method’s greenness with its potential application (Gałuszka et al. 2013; Mammana et al. 2022; Tobiszewski et al. 2014). Moreover, environment, health, and safety assessment (EHS) and life-cycle assessment (LCA) are two commonly used green evaluation methods that can be utilized as screening methods to identify possible risks of chemicals and to analyse chemical emissions into the environment (Tobiszewski et al. 2019).

### Comparative analysis of TOPSIS approach with green metrics tools

The comparison study was aimed at analysing correlations of the TOPSIS results with green metrics tools in an aggregated approach. Correlations for different metrics tools with TOPSIS are shown in Table 5. TOPSIS showed a significant, but negative correlation with AGREE metrics tool. The primary reason for this negative correlation is data variability between the range of ranking numbers and AGREE. In addition to the correlation matrix, which was based on metrics tool scores and TOPSIS ranking numbers, the methods were ranked in decreasing order of relative closeness, i.e., the method with the highest relative closeness value was ranked first. Except for ESA tool, there were only weak negative correlations between TOPSIS results and other metrics tools. The ESA tool was the only one that correlated positively with TOPSIS; however, it was a weak correlation. It should be noted, however, that the metrics tools considered in this work had relatively different range of scores and assessment categories as compared to TOPSIS ranking. NEMI and TOPSIS, for example, can be distinguished as two distinct cases of correlation outcomes. NEMI is the least informative tool because it only considers four parameters. The results of NEMI analysis can be natural numbers ranging from 0 to 4 based on the subcategories of the pictogram; however, they are only qualitative and provide no information about the quantity of hazards. TOPSIS ranking, on the other hand, considered twelve criteria, and the numerical values for each criterion were assigned any number between 0 and l.

**Table 5:** TOPSIS correlation matrix with metrics tools

	TOPSIS	NEMI	AGREE	GAPI	AGREEprep	ESA
TOPSIS	1					
NEMI	-0.06682	1				
AGREE	-0.85152	0.251533	1			
GAPI	-0.30632	-0.55621	0.41124	1		
AGREEprep	-0.03576	0.236449	0.182476	0.3691691	1	
ESA	0.116252	-0.21176	-0.24665	0.20682559	0.339908077	1

The wide variation in TOPSIS and NEMI scores and assessment categories contributed considerably to their low negative correlation. The obvious significant negative correlation between NEMI and GAPI is the results of their produced scores that are closer to each other, which improved their correlation. The lack of significant correlation between AGREE and NEMI, on the other hand, is not surprising, given that NEMI consistently produced high scores, reduced the size of the correlation between the two tools. The weak correlation between NEMI and AGREE, like the one between TOPSIS and NEMI, indicates that the range of scores between these tools varies significantly and is independent. However, it is worth noting that the highly ranked SPE-MEKC (method 7) with TOPSIS analysis was also highly scored with NEMI and AGREE tools. The scores of these tools are consistent with the greenness characteristics of the described SPE-MEKC. Additionally, TOPSIS algorithm allowed for the ranking of analytical procedures based on GAC criteria using AGREE scores as input data for the design matrix, which provided a significant advantage for the observed correlation between TOPSIS and AGREE tool.

## Conclusions

The purpose of this research was to select greener analytical method for mifepristone determination in water samples using TOPSIS approach, as well as to assess and profile the analytical methods’ greenness using the selected green metrics tools for the study. TOPSIS successfully enabled the effective selection of SPE-MEKC as an ideal green analytical procedure for mifepristone determination in environmental water samples. On the other hand, several assessment techniques for analysing the greenness of the described analytical methods were presented, including NEMI, Eco-Scale, GAPI, AGREE,and AGREEprep. Except for Eco-scale and NEMI for method 7, assessment studies of other metrics tools highlighted the need to develop new or appropriately modify existing analytical procedures, as no analytical procedure reported in this work is characterized by good environmental performance. In addition, comparative analysis of TOPSIS with metrics tools revealed that the AGREE tool correlated with TOPSIS, while other metrics tools had weak correlations. The use of AGREE metric scores, which directly link to the 12 GAC principles, to assign numerical values to criteria in the design of matrix for TOPSIS analysis was the key contribution of this work. This work was also able to report for the first time on the evaluation and profile of greenness assessment of the reported analytical procedures for mifepristone determination in environmental water samples.

In conclusion, the TOPSIS approach is relatively simple and effectively integrated GAC principles to select the most preferable analytical procedure with respect to greenness. The results of TOPSIS are easy to understand and allow for quick ranking of various analytical methodologies. TOPSIS, in combination with the metrics tools used, effectively offered a critical and objective assessment of the analytical procedures in terms of GAC principles. This current study should be expanded to include more diversified weighting approaches, additional criteria categories, and sensitivity testing.

### Supplementary Information

Below is the link to the electronic supplementary material.

Supplementary file1 (PDF 439 kb)

## Data Availability

Data will be made available upon request.
