# Supplementary material for: Multi-criteria decision analysis: technique for order of preference by similarity to ideal solution for selecting greener analytical method in the determination of mifepristone in environmental water samples
Source: Environ Sci Pollut Res Int. 2024 Apr 5;31(20):29460–71. doi: 10.1007/s11356-024-32961-3 (PMC11058867; doi:10.1007/s11356-024-32961-3)
Supplement: Supplementary file 1 — Supplementary file1 (PDF 439 kb) [file 11356_2024_32961_MOESM1_ESM.pdf]

Multi-criteria decision analysis–Technique for order of preference by similarity to ideal solution for selecting greener analytical method in the determination of mifepristone in environmental water samples.

Tlou Makwakwa [1,2], Elsie Moema [1], Titus Msagati [2 *]

[1]*Department of Chemistry, College of Science, Engineering and Technology, University of South Africa, Florida, 1709, Johannesburg, South Africa.*

[2]*Institute for Nanotechnology and Water Sustainability, College of Science, Engineering and Technology, University of South Africa, Florida, 1709, Johannesburg, South Africa.*

* *Corresponding author Email*: msagatam@unisa.ac.za, Tel +27 11 670 9482

Appendix A. Supplementary materials

The article's supplementary materials can be found online.

Supplementary Data

Multi-criteria decision analysis–Technique for order of preference by similarity to ideal solution for selecting greener analytical method in the determination of mifepristone in environmental water samples.

Tlou Makwakwa [1,2], Elsie Moema [1], Titus Msagati [2 *]

[1]*Department of Chemistry, College of Science, Engineering and Technology, University of South Africa, Florida, 1709, Johannesburg, South Africa.*

[2]*Institute for Nanotechnology and Water Sustainability, College of Science, Engineering and Technology, University of South Africa, Florida, 1709, Johannesburg, South Africa.*

* *Corresponding author Email*: msagatam@unisa.ac.za, Tel +27 11 670 9482

TOPSIS approach

The mean weight objective weighting method was used in this investigation**.** The mean weight (MW) was calculated using equation (1) and assumes that all criteria are equally important:

$$w_j = \frac{1}{n}; \text{where n is the number of criteria} \quad (1)$$

The TOPSIS application was separated into the following steps:

The creation of a normalized decision matrix

$$r_{ij} = x_{ij} \div \sqrt{\sum x_{ij}^2},\ i = 1, 2, \ldots, m \text{ and } j = 1, 2, \ldots, n, \quad (2)$$

where x_{ij} and r_{ij} are original and normalized scores in decision matrix.

Construction of the weighted normalised decision matrix

$$V_{ij} = r_{ij} \times W_j,\ i = 1, 2, \ldots, m \text{ and } j = 1, 2, \ldots, n, \quad (3)$$

where w_j is the weight of the criterion j and $\sum_{j=1}^{n} = 1$

Determination of positive ideal (A*) and negative (A$^-$) solutions

$$A^* = \{(\max_i v_{ij} \mid j \in C_b), (\min_i V_{ij} \mid j \in C_c)\} = \{V_i^* \mid j = 1, 2, \ldots, m\} \quad (4)$$

$$A^- = \{(\min_i v_{ij} \mid j \in C_c), (\max_i V_{ij} \mid j \in C_c)\} = \{V_j^* \mid j = 1, 2, \ldots, m\} \quad (5)$$

Calculation of the separation measures for each relative

$$S_i^* = \sqrt{\sum_{j=1}^{m}(V_{ij} - V_j^*)^2};\ j = 1, 2, \ldots, m \quad (6)$$

$$S_i^- = \sqrt{\sum_{j=1}^{m}(V_{ij} - V_j^-)^2};\ j = 1, 2, \ldots, m \quad (7)$$

Calculation of relative closeness to the ideal solution

$$C_i^* = \frac{S_i^-}{S_i^* + S_i^-},\ i = 1, 2, \ldots, m \text{ and } 0 < C_i^* < 1 \quad (8)$$

Table S1. TOPSIS decision matrix based on the 12 GAC principles.

Method No.	Criteria 1	Criteria 2	Criteria 3	Criteria 4	Criteria 5	Criteria 6	Criteria 7	Criteria 8	Criteria 9	Criteria 10	Criteria 11	Criteria 12
1	0.48	0.0	0.0	0.8	0.5	1.0	0.45	0.8	0.0	0.0	0.26	0.8
2	0.48	0.0	0.0	0.8	0.5	1.0	0.49	0.64	0.0	0.0	0.3	0.8
3	0.48	0.0	0.0	0.8	0.5	1.0	0.49	0.64	0.0	0.0	0.3	0.8
4	0.48	0.0	0.0	0.8	0.0	1.0	0.45	0.95	0.0	0.0	0.27	0.8
5	0.6	0.0	0.33	1.0	0.0	1.0	0.08	0.05	1.0	0.0	0.15	0.8
6	0.48	0.0	0.0	0.8	0.0	1.0	0.55	0.53	0.0	0.0	0.36	0.8
7	0.48	0.0	0.0	0.8	0.0	1.0	0.69	0.34	1.0	0.5	0.5	0.8
8	0.48	0.0	0.0	0.8	0.0	1.0	0.42	0.69	0.0	0.0	0.24	0.8
9	0.48	0.0	0.0	0.8	0.0	1.0	0.45	0.8	0.0	0.0	0.26	0.8
10	0.48	0.0	0.0	0.8	0.0	1.0	0.43	0.84	0.0	0.0	0.25	0.8
11	0.48	0.0	0.0	0.8	0.0	1.0	0.42	0.69	0.0	0.0	0.24	0.8
12	0.48	0.0	0.0	0.8	0.0	1.0	0.42	0.82	0.0	0.0	0.24	0.8
13	0.48	0.0	0.0	0.8	0.0	1.0	0.42	0.95	0.0	0.0	0.24	0.8
